# Interaction of the Fungal Metabolite Harzianic Acid with Rare-Earth Cations (Pr^3+^, Eu^3+^, Ho^3+^, Tm^3+^)

**DOI:** 10.3390/molecules27196468

**Published:** 2022-10-01

**Authors:** Maria Michela Salvatore, Antonietta Siciliano, Alessia Staropoli, Francesco Vinale, Rosario Nicoletti, Marina DellaGreca, Marco Guida, Francesco Salvatore, Mauro Iuliano, Anna Andolfi, Gaetano De Tommaso

**Affiliations:** 1 Department of Chemical Sciences, University of Naples Federico II, 80126 Naples, Italy; 2 Institute for Sustainable Plant Protection, National Research Council, 80055 Portici, Italy; 3 Department of Biology, University of Naples Federico II, 80126 Naples, Italy; 4 Department of Agricultural Sciences, University of Naples Federico II, 80055 Portici, Italy; 5 Department of Veterinary Medicine and Animal Productions, University of Naples Federico II, 80137 Naples, Italy; 6 BAT Center-Interuniversity Center for Studies on Bioinspired Agro-Environmental Technology, University of Naples Federico II, 80055 Portici, Italy; 7 Council for Agricultural Research and Economics, Research Centre for Olive, Fruit and Citrus Crops, 81100 Caserta, Italy

**Keywords:** rare-earth elements, lanthanides, harzianic acid, organic ligands, fungal metabolites, *trichoderma*, rare-earth ecotoxicology

## Abstract

Rare-earth elements (REEs) are in all respect a class of new contaminants that may have toxic effects on organisms and microorganisms and information on their interactions with natural ligands should be of value to predict and control their diffusion in natural environments. In the current study, we investigate interactions of tripositive cations of praseodymium, europium, holmium, and thulium with harzianic acid (H_2_L), a secondary metabolite produced by selected strains of fungi belonging to the *Trichoderma* genus. We applied the same techniques and workflow previously employed in an analogous study concerning lanthanum, neodymium, samarium, and gadolinium tripositive cations. Therefore, in the current study, HPLC-ESI-HRMS experiments, circular dichroism (CD), and UV-Vis spectrophotometric absorption data, as well as accurate pH measurements, were applied to characterize bonding interactions between harzianic acid and Pr^3+^, Eu^3+^, Ho^3+^, and Tm^3+^ cations. Problems connected to the low solubility of harzianic acid in water were overcome by employing a 0.1 M NaClO_4_/(CH_3_OH + H_2_O 50/50 *w*/*w*) mixed solvent. For Pr^3+^, Ho^3+^, and Tm^3+^, only the mono complexes PrL^+^, HoL^+^, and TmL^+^ were detected and their formation constant determined. Eu^3+^ forms almost exclusively the bis complex ${\mathrm{EuL}}_{2}^{-}$ for which the corresponding formation constant is reported; under our experimental conditions, the mono complex EuL^+^ is irrelevant. Combining the results of the present and previous studies, a picture of interactions of harzianic acid with rare-earth cations extending over 8 of the 17 REEs can be composed. In order to complement chemical information with toxicological information, a battery of bioassays was applied to evaluate the effects of praseodymium, europium, holmium, and thulium tripositive cations on a suite of bioindicators including *Aliivibrio fischeri* (Gram-negative bacterium), *Raphidocelis subcapitata* (green alga), and *Daphnia magna* (microcrustacean), and median effective concentration (EC50) values of Pr^3+^, Eu^3+^, Ho^3+^, and Tm^3+^ for the tested species were assessed.

## 1. Introduction

Rare-earth elements (REEs) are extensively used in several anthropogenic activities due to a spectrum of unique and valuable physicochemical properties. REEs are considered excellent electrical conductors and are employed in various high-technology industrial products. For example, rare-earth elements and compounds are used in the production of contrast agents in medical diagnostics, anti-tumor drugs, electrical and electronic devices and equipment, luminescent materials, high-performance lightweight permanent magnets, solar panels, and wind turbines [1,2,3,4]. Furthermore, in recent years, low doses of rare-earth elements have also been used in agriculture in fertilizers and as livestock growth promoters [5,6,7].

Obviously, because of their widespread application in high-tech manufacturing industries worldwide as well as in agriculture and of the subsequent release into soil, water, and atmosphere, the risk of uncontrolled human exposure to REEs is progressively growing [8]. Among others, contamination of water bodies by rare-earth elements is a particularly hot issue since ingestion of contaminated aquatic foods and drinking water are primary pathways for human exposure to REEs and an increasing body of literature has reported, from time to time, anthropogenic rare-earth elements in aquatic systems in various countries [9,10]. For example, high levels (in the ng L^−1^ range) of gadolinium, from gadolinium-based contrast agents, have been frequently documented in aquatic environments in developed countries, and lanthanum and samarium have been detected in the river Rhine at alarmingly high concentrations [11]. Therefore, REEs are considered in all respects a new category of emerging contaminants, and public awareness and concern are gradually increasing [3,12,13,14,15].

Fungi and plants play an important role in the bioavailability of REEs through the production of secondary metabolites which in general are potential ligands of rare-earth cations capable of influencing their mobilization in the environment [16]. In fact, studies of the coordination chemistry in aqueous solutions of natural organic ligands with rare-earth cations are steadily accelerating and are increasingly important for understanding their effect on organisms and microorganisms [17,18,19,20].

A recent study indicated that fungi of the genus *Trichoderma* tolerate the presence of REEs in the culture media [21], and this may be due to the masking of rare-earth cations by complex formation with fungal metabolites. In fact, *Trichoderma* spp. are well-known producers of compounds showing intriguing chemical and biological properties, such as antimicrobial [22,23,24,25], antibiofilm [26], antiproliferative [23], and plant growth promoting activities [27,28].

Lately, we have undertaken studies to evaluate the sequestering capabilities of the *Trichoderma* metabolite harzianic acid toward a variety of metal cations [29,30], including a selection of tripositive rare-earth cations, i.e., La^3+^, Nd^3+^, Sm^3+^, and Gd^3+^ [31]. Remarkably, it was observed that harzianic acid is an efficient ligand for these four rare-earth cations. Furthermore, by applying a bioassay battery, we first demonstrated that Gd^3+^ is highly toxic to several bioindicator organisms, and then that Gd^3+^ toxicity decreases in presence of harzianic acid presumably because of the capacity of this metabolite of masking rare-earth cations by complex formation [31]. Hence, the encouraging results obtained for the above-listed rare-earth cations stimulated us to employ the same experimental workflow to collect data on the coordination properties of harzianic acid toward tripositive cations of four additional less-studied lanthanides, i.e., praseodymium, europium, holmium, and thulium tripositive cations.

High-resolution mass spectra (HRMS), acquired by HPLC-ESI-TOF, provided general evidence of the capability of harzianic acid to interact with the targeted rare-earth cations (i.e., Pr^3+^, Eu^3+^, Ho^3+^, and Tm^3+^). Subsequently, in order to obtain a qualitative and quantitative view of complex formation equilibria in solution, CD and UV-Vis spectra spanning a wide wavelength range were acquired at 25 °C on a suite of solutions of accurately known pH and analytical concentrations of each of the targeted rare-earth cations and harzianic acid. As in our previous work [31], because of the low solubility of harzianic acid in the water, the stoichiometry and complex formation constants of the selected lanthanides cations with harzianic acid were evaluated in a 0.1 M NaClO_4_/(CH_3_OH + H_2_O 50/50 *w*/*w*) mixed solvent.

Since risk assessment of exposure to REEs in the environment is an urgent need, a battery of ecotoxicological tests was used to evaluate the effect of praseodymium, europium, holmium, and thulium tripositive cations on a set of bioindicators representative of different levels of biological complexity and trophic levels including *Aliivibrio fischeri* (Gram-negative bacterium), *Raphidocelis subcapitata* (green alga), and *Daphnia magna* (microcrustacean). In this respect, to our knowledge, this is the first paper reporting median effective concentration (EC50) of Pr^3+^, Eu^3+^, Ho^3+^, and Tm^3+^ for *D. magna, R. subcapitata*, and *A. fischeri*.

## 2. Results and Discussion

### 2.1. Harzianic Acid

**Figure 1:**
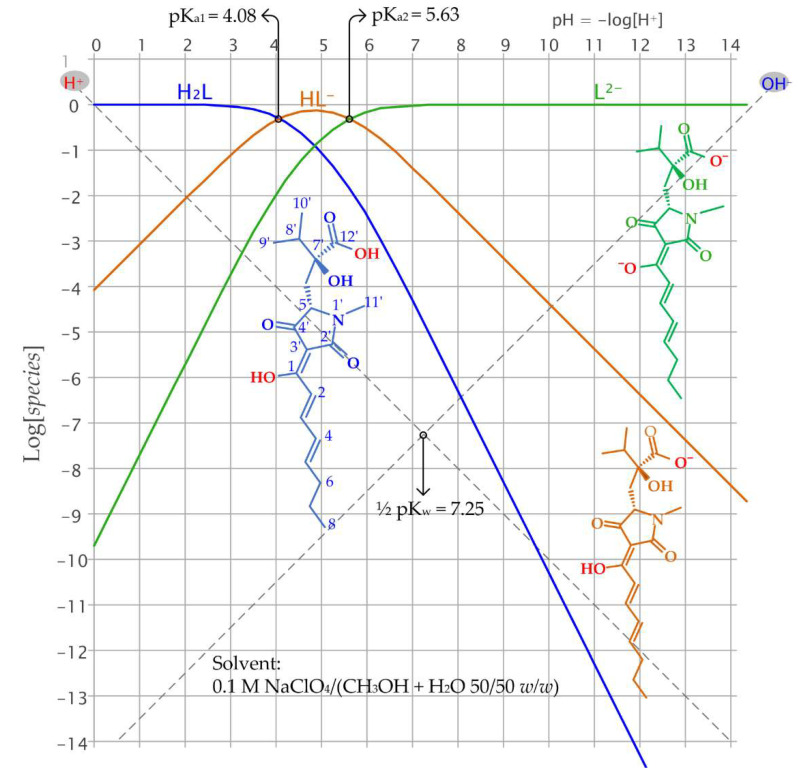
Logarithmic acid-base diagram of harzianic acid (H_2_L) at a conventional analytical concentration of 1 M. Reported dissociation constants of harzianic acid (*K*_a1_ = 10^−4.08^ and *K*_a2_ = 10^−5.63^) and the ionic product of water (*K*_w_ = 10^−14.5^) were determined at 25 °C in the mixed solvent 0.1 M NaClO_4_/(CH_3_OH + H_2_O 50/50 *w*/*w*) [29]. Inserts: structures of harzianic acid (H_2_L) and its dissociation products (HL^−^ and L^2−^).

Harzianic acid is a diprotic acid, from this point forward indicated with the abbreviated formula H_2_L. The logarithmic acid-base diagram in Figure 1, at a conventional analytical concentration of harzianic acid of 1 M, provides an easy insight into its acid-base properties displaying how the equilibrium concentrations of the three species H_2_L, HL^−^, and L^2−^ depend on pH (= −log[H^+^]).

The fully protonated species, H_2_L, is the prevailing species at pH < 4.08 (= pK_a1_), and the fully deprotonated L^2−^ species predominates at pH > 5.63 (= pKa_2_). The equilibrium concentration of the amphiprotic HL^−^ species rises over the equilibrium concentrations of H_2_L and L^2−^ in the narrow range of pH between pK_a1_ and pK_a2_. Hence, the fully deprotonated species (L^2−^) prevails over the monoprotonated (HL^-^) and fully protonated (H_2_L) forms at the typical pH of biological and natural systems [29]. Because protonation of the binding sites of ligands which are weak acids is a collateral reaction that may prevent bonding to metal cations, the prevalence of the L^2−^ species at relatively low pH is an important factor contributing to the efficiency of harzianic acid as a ligand for metal cations in natural environments.

Because harzianic acid has low solubility in water, the acid dissociation constants reported in Figure 1 have been determined, at 25 °C, in a 0.1 M NaClO_4_/(CH_3_OH + H_2_O 50/50 *w*/*w*) solvent which is also the solvent employed for the investigation of complex formation equilibria between harzianic acid and the targeted lanthanide cations (i.e., Pr^3+^, Eu^3+^, Ho^3+^, and Tm^3+^).

### 2.2. Coordination Properties of Harzianic Acid toward Pr^3+^, Eu^3+^, Ho^3+^, and Tm^3+^

Complex formation equilibria between harzianic acid and the tripositive rare-earth cations Pr^3+^, Eu^3+^, Ho^3+^, and Tm^3+^ were studied by applying mass spectrometric, potentiometric, and spectrophotometric techniques. For convenience, the general symbol Ln^3+^ is employed in subsequent sections to indicate the four tripositive rare-earth cations under examination.

First, HPLC-ESI-TOF was employed to acquire high-resolution mass spectra (HRMS) on solutions prepared by mixing 500 µL of 2 mM aqueous solution of the chloride or perchlorate salts of each Ln^3+^ cation and 500 µL of harzianic acid 1 mg mL^−1^ in methanol. Table 1 shows the most abundant ions in the collected mass spectra.

It can be seen that all spectra expose well-developed mass peaks corresponding to the adduct ions of harzianic acid with hydrogen (i.e.,$\;{\left[H_{2}L+H\right]}^{+}$, *m/z* 366.1921) and with sodium (i.e., ${\left[H_{2}L+\mathrm{Na}\right]}^{+}$, *m/z* 388.1730). Furthermore, mass peaks which can be attributed to ions containing both harzianic acid and the Ln^3+^ cations (i.e., ${\left[2H_{2}L-2H+\mathrm{Ln}\right]}^{+}\equiv \mathrm{Ln}{\left(\mathrm{HL}\right)}_{2}^{+}$) are also observed. This is a useful preliminary indication of the capability of harzianic acid to interact with the considered rare-earth cations.

In order to obtain a qualitative and quantitative view of interactions between harzianic acid and Ln^3+^ cations in solution, CD and UV-Vis spectra spanning a wide wavelength range (~200–~500 nm) were acquired, at 25 °C, on solutions of accurately known analytical concentrations of the metal cation ($C_{\mathrm{Ln}}\;M$) and of the ligand ($C_{H_{2}L}\;M$) in a 0.1 M NaClO_4_/(CH_3_OH + H_2_O 50/50 *w*/*w*) solvent and which “pH” was measured with a glass indicator electrode. Since, as described in the Materials and Methods section, the glass electrode was calibrated to respond to the molar free proton concentration, [H^+^], in the following the conventional symbol “pH” merely indicates the antilogarithm of the molar free proton concentration in the investigated solutions (i.e., we define pH = −log[H^+^]). Altogether, CD and UV-Vis spectra were acquired on each of the 48 solutions exposed in Table 2.

As can be seen from Table 2, for each rare-earth cation, two groups of CD and UV-Vis spectra were acquired which are distinguished by their ligand-to-metal ratio (i.e., $C_{H_{2}L}/C_{\mathrm{Ln}}$). The first group of spectra had a constant $C_{H_{2}L}/C_{\mathrm{Ln}}≅1$ ratio, whereas the second group of spectra had $C_{H_{2}L}/C_{\mathrm{Ln}}≅2$. For each rare-earth cation, the exact $C_{H_{2}L}/C_{\mathrm{Ln}}$ ratio in each of the two groups of spectra is given in the second column of Table 2. Test solutions to be presented to the CD and UV-Vis spectrophotometers were prepared by implementing an accurately planned preparation protocol described in detail in the Material and Methods section. The preparation protocol allowed us to maintain the same $C_{H_{2}L}/C_{\mathrm{Ln}}$ ratio through test solutions belonging to the same group and, most importantly, allowed to integrate the calibration of the glass electrode into the preparation procedure which is essential for achieving maximum accuracy in the measurement of the pH of each test solution. This acquisition strategy may be useful because it can help in the interpretation of data; in fact, a comparison of spectra acquired at different pH but having the same ligand-to-metal ratio could be easier and more informative with respect to the alternative option of having the concentrations of the metal cation and the ligand changing wildly in the investigated solutions.

**Figure 2:**
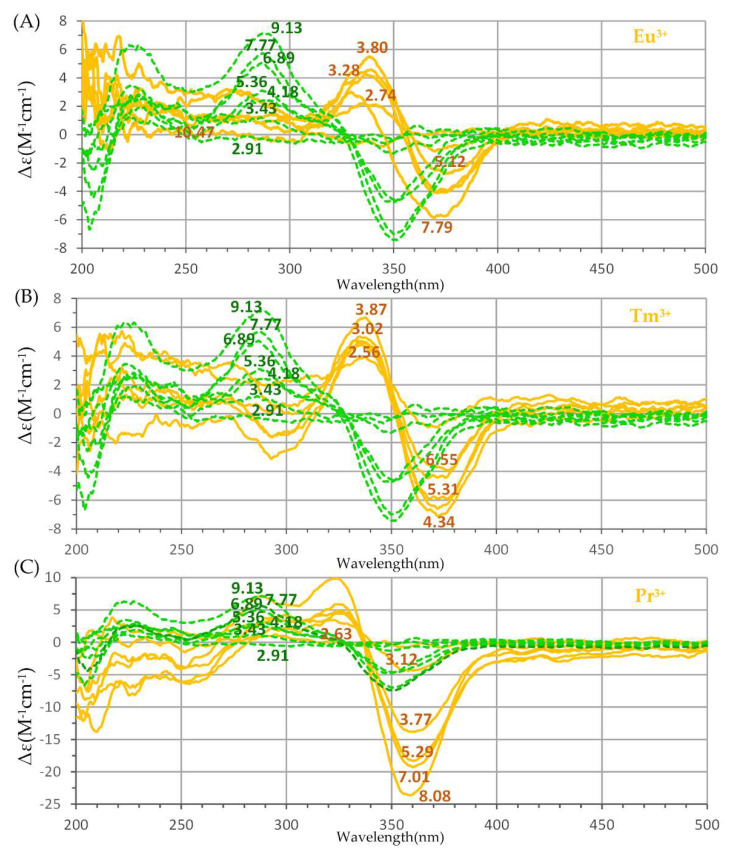
(**A**) Superimposition of Far–UV circular dichroism (CD) spectra of harzianic acid (≤1.72 × 10^−4^ M) at different pH (green dashed curves) and of CD spectra at different pH of: (**A**) Eu^3+^-harzianic acid system at 1.044: 1 ligand-to-metal ratio (full orange curves); (**B**) Tm^3+^-harzianic acid system at 1.037: 1 ligand-to-metal ratio (full orange curves); (**C**) Pr^3+^-harzianic acid system at 1.978: 1 ligand-to-metal ratio (full orange curves). All spectra have been acquired in a 0.1 M NaClO_4_/(CH_3_OH + H_2_O 50/50 *w*/*w*) solvent. Numerical labels on curves indicate the corresponding pH and may serve as a link to connect each spectrum to solutions in Table 2.

In Figure 2, CD spectra of a few Ln^3+^-harzianic acid solutions from Table 2 (full orange curves) are compared to CD spectra of solutions of harzianic acid (green dashed curves) in the 0.1 M NaClO_4_/(CH_3_OH + H_2_O 50/50 *w*/*w*) solvent.

**Figure 3:**
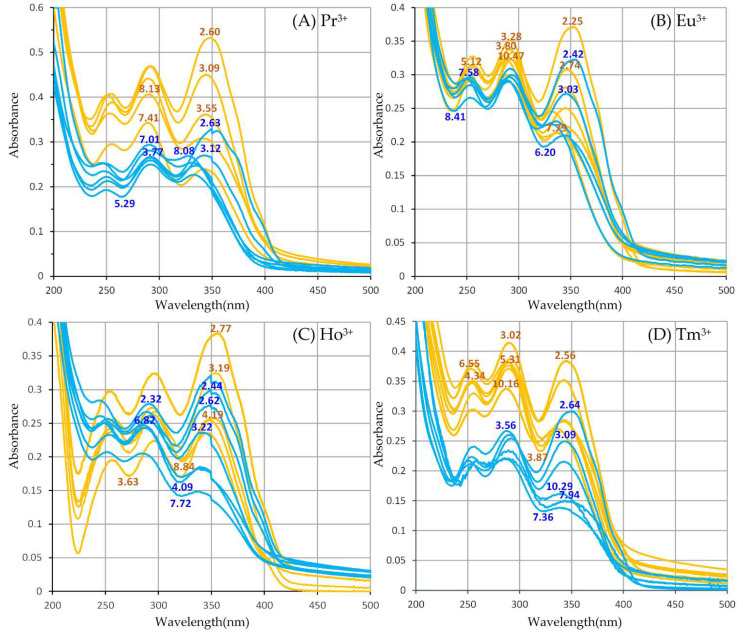
Raw UV-Vis spectra of (**A**) solutions of Pr^3+^ and harzianic acid, (**B**) solutions of Eu^3+^ and harzianic acid, (**C**) solutions of Ho^3+^ and harzianic acid, and (**D**) solutions of Tm^3+^ and harzianic acid of accurately known analytical composition and pH (= −log[H^+^]). For each metal cation, absorption spectra define two groups differentiated by color: orange-colored spectra have been acquired on solutions with a nearly equal concentration of the ligand and metal; blue spectra have been acquired on solutions with a concentration of the ligand nearly twice that of the metal cation. Numerical labels on curves indicate the corresponding pH. Details on ligand and metal concentrations employed to collect the UV-Vis spectra are reported in Table 2. pH can be used to connect each spectrum to the corresponding solution in Table 2.

CD spectra of solutions of harzianic acid are characterized by a negative Cotton effect with a positive peak at about 280 nm and a negative peak at about 350 nm and expose a strong dependence on pH (indicated by the numerical label on each spectrum) which governs the equilibrium concentrations of the H_2_L, HL^−^, and L^2−^ species in solution. The dramatic change introduced in CD spectra of harzianic acid by the presence of the Ln^3+^ cations is very likely the result of complex formation reactions, creating species that are not present in solutions of harzianic acid only. On the other side, the entity of the alterations observed in the CD spectra of harzianic acid depends on the specific Ln^3+^ cation and on the ligand-to-metal ratio (e.g., compare Figure 3C with Figure 3A,B). This is very likely the result of differences in the stoichiometry and/or formation constants of Ln^3+^/harzianic acid complexes.

UV-Vis spectra corresponding to solutions of Ln^3+^ cations and harzianic acid in Table 2 are presented in Figure 3.

In Figure 3, for each Ln^3+^ cation, orange-colored spectra correspond to solutions in the $C_{H_{2}L}/C_{\mathrm{Ln}}≅1$ group, whereas blue-colored spectra correspond to solutions in the $C_{H_{2}L}/C_{\mathrm{Ln}}≅2$ group. As for CD spectra in Figure 2, the numerical label associated to each UV-Vis spectrum indicates the pH of the solution on which the spectrum was acquired and can be used as a link to the corresponding solution in Table 2.

In order to evaluate the stoichiometry and formation constants of Ln^3+^/harzianic acid complexes eventually present in the investigated solutions, UV-Vis spectra in Figure 3 were submitted to the well-known Hyperquad program [32,33]. To this end, for each Ln^3+^ metal cation, a matrix (Abs matrix) is created containing the measured UV-Vis spectra in Figure 3. One spectrum is assigned to column k of the Abs matrix and is represented by a collection of discrete absorbance values $A_{ik}$ measured at the wavelength specified by the row index, i. Four Abs matrices are created (one for each Ln^3+^ cation) which are presented, one by one, to the Hyperquad program together with the pertinent metadata specifying the analytical composition and pH of solutions on which each spectrum was acquired (and which are linked to the column index, *k*, of the Abs matrix).

However, in order to run the Hyperquad program on a given Abs matrix the user must also provide a speciation model by specifying the stoichiometry of the species present in the solution. In fact, the function of the Hyperquad program is merely that of evaluating the entered speciation model by systematically modifying the formation constants and molar extinction coefficients of the postulated species to achieve the minimum of the sum of the squared residuals, U, as defined by Equation (1):

(1) $$U=\sum_{i}\sum_{k}{\left(A_{ik}-A_{ik}^{c}\right)}^{2}$$

In Equation (1), $A_{ik}$ represents the measured absorbance in row i and column k of the Abs matrix and $A_{ik}^{c}$ is the corresponding absorbance calculated by the program on the basis of the entered model and analytical composition and pH of each solution. Once a minimum value of U has been found, Hyperquad outputs the formation constants of species in solution which produce, for the evaluated model, the best fit of the experimental data and corresponding estimated standard deviations.

In the present case, formation constants in the output of the Hyperquad program are indicated as $\beta_{pqr}$ since formally each species in the investigated solutions is supposed to be formed through the general reaction (2):

(2) $$p{\mathrm{Ln}}^{3+}+qL^{2-}+rH^{+}⇋{\mathrm{Ln}}_{p}L_{q}H_{r}^{\left(3p-2q+r\right)+}\;\beta_{pqr}=\frac{\left[{\mathrm{Ln}}_{p}L_{q}H_{r}^{\left(3p-2q+r\right)+}\right]}{{\left[{\mathrm{Ln}}^{3+}\right]}^{p}[L^{2-}{]}^{q}[H^{+}{]}^{r}}\;$$

**Figure 4:**
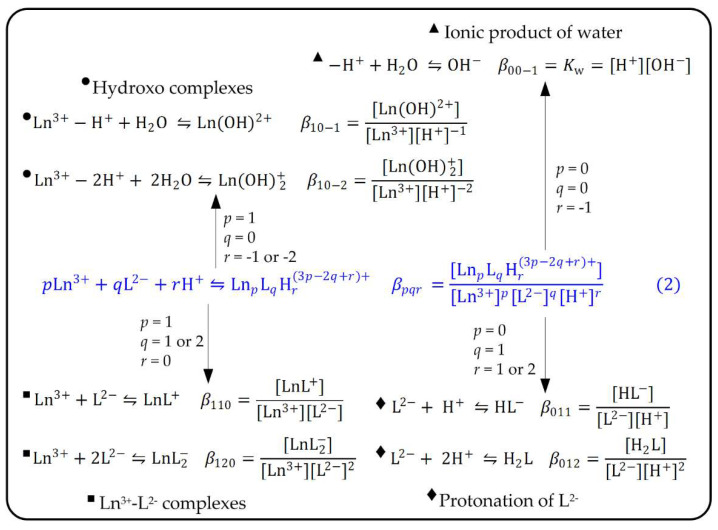
Examples of instances of the general Equation (2) employed to generate a speciation model to be evaluated by the Hyperquad program and significance of the $\beta_{pqr}$ formation constants in the output of the program.

In fact, a speciation model is simply a collection of instances of reaction (2) obtained by specifying a set of (*p*, *q r*) coefficients. For example, a hypothetical model comprising the ${\mathrm{LnL}}^{+}$ and ${\mathrm{LnL}}_{2}^{-}$ complexes of harzianic acid with Ln^3+^ cations, the hydroxo complexes ${\mathrm{LnOH}}^{2+}$ and $\mathrm{Ln}{\left(\mathrm{OH}\right)}_{\;2}^{+}$ and the L^2−^, ${\mathrm{HL}}^{-}$ and $H_{2}L$ species is fully specified by the instances of reaction (2) and corresponding $\beta_{pqr}$ formation constants collected in Figure 4.

The challenge with the Hyperquad program is that it will neither accept nor reject the submitted speciation model but will simply output the achieved minimum of the sum of squared residuals, U, and corresponding values of the $\beta_{pqr}$ formation constants defined by the model. The implication of this is that the program must be run on a given Abs matrix many times assuming different speciation models and the values of U corresponding to different models must be compared. In abstract, the correct model is the one that produces the lowest value of U. In practice, this recursive process is very slippery because of the tendency of U to assume very similar values with different models or to decrease below what can be expected from the experimental error by increasing the number of species included in the model.

A maximum parsimony criterion, according to which the correct model is the one that explains the data within experimental error by assuming the minimum number of species is a valuable guide in the process of assessing the stoichiometry of complex species actually present in the examined solutions.

A second guiding principle, very useful for avoiding overfitting of the experimental data and the introduction of artifacts in the model, can be deduced by inspecting Figure 4. We see that in the model submitted to Hyperquad there are a few reactions and equilibrium constants that can be evaluated separately in a simpler experimental setting and kept unchanged during the whole recursive process of minimization of U. In particular, the protonation constants of the ligand (i.e., $\beta_{011}$ = 10^5.63^ and $\beta_{012}\;$= 10^9.71^) can be determined separately by measurements on solutions that do not contain the metal cation, and the ionic product of water, $K_{w}=$ 10^−14.5^, in the 0.1 M NaClO_4_/(CH_3_OH + H_2_O 50/50 *w*/*w*) solvent, can obviously be evaluated from potentiometric strong acid-strong base titrations which neither involve the ligand nor the metal cation.

On the other side, the number and stoichiometry of hydroxo complexes to be introduced in the models can be estimated from literature data pertaining to the hydrolysis of the investigated cations. On this basis, all models submitted to the Hyperquad program in this study included only the $\mathrm{Ln}{\left(\mathrm{OH}\right)}^{2+}$ hydroxo complex which, in water, is by far the prevailing species in the pH range and at the concentration of rare-earth cations investigated [34].

Results of Hyperquad processing of spectrophotometric data in Figure 3 are summarized in Table 3.

The presented results were obtained by leveraging on the maximum parsimony criterion presented above.

In the case of Pr^3+^, Ho^3+^, and Tm^3+^ a model including, both the ${\mathrm{LnL}}^{+}$ mono complex and the bis complex, ${\mathrm{LnL}}_{2}^{-}$, did not improve appreciably the sum of squared residuals U with respect to a model which included only the formation of the mono complex. Therefore, it was finally assumed that under the present experimental conditions only mono complexes of Pr^3+^, Ho^3+^, and Tm^3+^ with harzianic acid are formed according to the $\beta_{110}$ formation constants in Table 3.

In the case of Eu^3+^ we had to deal with the opposite dilemma since about the same value of the sum of squared residuals U was obtained either from a model including both ${\mathrm{EuL}}^{+}$ and ${\mathrm{EuL}}_{2}^{-}$ complexes or from a model including only the ${\mathrm{EuL}}_{2}^{-}$ bis complex. Even in this case we have assumed the model including only the ${\mathrm{EuL}}_{2}^{-}$ bis complex whose $\beta_{120}$ formation constant is reported in Table 3. However, we also report an estimate of the upper limit of the formation constant, $\beta_{110}$, of the ${\mathrm{EuL}}^{+}$ mono complex compatible with our data (i.e., we do not exclude that the ${\mathrm{EuL}}^{+}$ complex is formed but exclude that its formation constant is larger than the value reported in Table 3).

**Figure 5:**
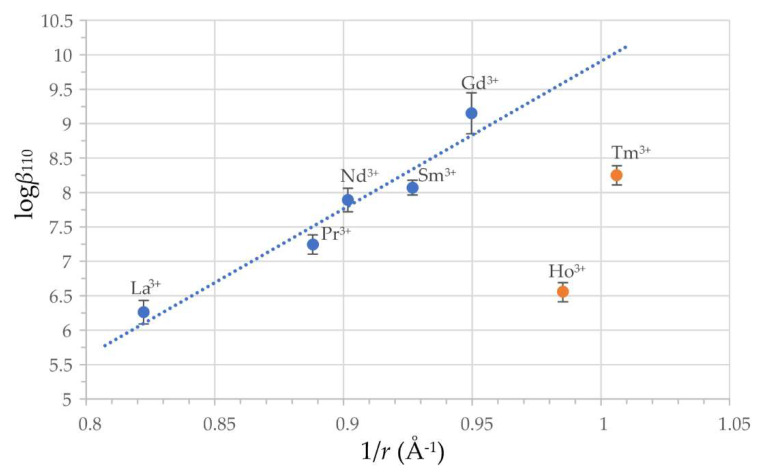
Correlation of formation constants *β*_110_ for reaction Ln^3+^ + L^2−^ ⇋ LnL^+^ from Table 3 and reference [31] with the reciprocal of ionic radius (i.e., $r^{-1}$) of Ln^3+^ cations: La^3+^ (*r* = 1.216 Å), Pr^3+^ (*r* = 1.126 Å), Nd^3+^ (*r* = 1.109 Å), Sm^3+^ (*r* = 1.079 Å), Gd^3+^ (*r* = 1.053 Å), Ho^3+^ (*r* = 1.015 Å), Tm^3+^ (*r* = 0.994 Å) [34]. Dotted line is a least squares regression line (R^2^ = 0.94), trough points representing La^3+^, Pr^3+^, Nd^3+^, Sm^3+^, and Gd^3+^ (blue dots) and error bars represent three-times the estimated standard deviation (3*σ*) of log*β*_110_. Orange dots representing Ho^3+^ and Tm^3+^ are considered outliers.

In our previous investigation of complex formation equilibria of La^3+^, Nd^3+^, Sm^3+^, and Gd^3+^ cations with harzianic acid we found that, to an excellent approximation, the logarithm of the formation constants of their mono complexes (i.e., log$\beta_{110}$) increases linearly with the reciprocal of the ionic radius ($r^{-1}$). In Figure 5 we see that the log$\beta_{110}$ determined in this work for Pr^3+^ fits beautifully in this picture (the determination coefficient for the regression line in Figure 5 is R^2^ = 0.94). However, the linear increase in log$\beta_{110}$ with $r^{-1}$ does not continue indefinitely but stops in the correspondence of gadolinium since the formation constants of ${\mathrm{HoL}}^{+}$ and ${\mathrm{TmL}}^{+}$ are much lower than expected based on their ionic radius and the regression line in Figure 5.

**Figure 6:**
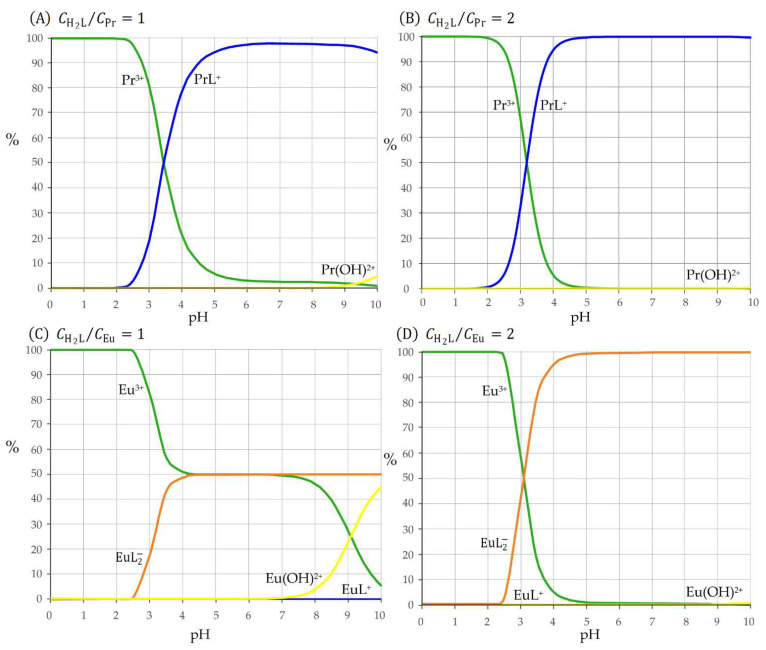
Distribution diagrams for: Pr^3+^–harzianic acid and Eu^3+^–harzianic acid systems: (**A**,**C**) equal total concentrations (10^−4^ M) of harzianic acid and metal cations; (**B**,**D**) harzianic acid total concentration (2 × 10^−4^ M) twice the total concentration of metal cations.

The distribution diagrams in Figure 6A,B disclose the chemistry which takes place in solutions containing Pr^3+^ and harzianic acid. It can be seen that at low pH the prevailing species in the solution is the free rare-earth cation, Pr^3+^, because protonation reactions inactivate the ligand bonding sites and prevent the complex formation reaction. As the pH of the solution increases, the effect of the collateral protonation reactions of the ligand fades out and the fraction of Pr^3+^ present in the form of the ${\mathrm{PrL}}^{+}$ mono complex readily rises to ~100% either when the $C_{H_{2}L}/C_{\mathrm{Pr}}=1$ as in Figure 6A or when $C_{H_{2}L}/C_{\mathrm{Pr}}=2$ as in Figure 6B. With minor variations due to changes in the value of the $\beta_{110}$ formation constant, these considerations can be extended to Ho^3+^/harzianic acid and Tm^3+^/harzianic acid systems.

However, under the same conditions, the scenario changes dramatically in solutions containing Eu^3+^ and harzianic acid. Either in solutions containing equal concentrations of the cation and of the ligand (i.e., $C_{H_{2}L}/C_{\mathrm{Eu}}=1$, Figure 6C) or in solutions in which the ligand concentration is twice the concentration of the cation (i.e., $C_{H_{2}L}/C_{\mathrm{Eu}}=2$, Figure 6D) as the pH increases the europium cation is converted to the bis complex ${\mathrm{EuL}}_{2}^{-}$. The fraction of Eu^3+^ present in the form of the ${\mathrm{EuL}}_{2}^{-}$ readily rises to 100% only in solutions in which $C_{H_{2}L}/C_{\mathrm{Eu}}≧2$ because when $C_{H_{2}L}/C_{\mathrm{Eu}}<2$ the ligand in the solution is stoichiometrically insufficient for the complete conversion of Eu^3+^ cation to the ${\mathrm{EuL}}_{2}^{-}$ bis complex. For instance, in Figure 6C, the fraction of europium complexed by harzianic acid rises only up to 50% which is the maximum value that can be achieved in a solution with an equal concentration of Eu^3+^ and harzianic acid. Although distribution diagrams in Figure 6C,D have been drawn by using the upper limit of the ${\mathrm{EuL}}^{+}$ complex formation constant in Table 3, the fraction of europium present as the mono complex is always very low, so much so that ${\mathrm{EuL}}^{+}$ can be ignored. Qualitatively, speciation of Eu^3+^ cation in harzianic acid solutions is like to the speciation of La^3+^ described in our previous work [31] since in both cases the bis complex seems to be formed in a single step directly from the La^3+^ or Eu^3+^ cation and the ligand (L^2−^).

Although by simply comparing the formation constants in Table 3 we immediately see that the efficiency of harzianic acid as a ligand declines from Tm^3+^ ($\beta_{110}$= 10^8.25^) to Pr^3+^ $\beta_{110}$ = 10^7.20^) and then from Pr^3+^ to Ho^3+^ ($\beta_{110}$ = 10^6.59^), it is not immediately evident if harzianic acid must be considered a more effective ligand for Eu^3+^ or for the other Ln^3+^ cations, simply because the $\beta_{110}$ formation constants of ${\mathrm{TmL}}^{+}$, ${\mathrm{PrL}}^{+}$, and ${\mathrm{HoL}}^{+}$ complexes cannot be compared to the $\beta_{120}$ formation constant of the ${\mathrm{EuL}}_{2}^{-}$ complex. To solve this dilemma, we can resort to the pM calculation for Eu^3+^-harzianic acid, Tm^3+^-harzianic acid, Pr^3+^-harzianic acid, and Ho^3+^-harzianic acid systems.

The pM is a conventional parameter, which can be associated with a metal cation-ligand system, defined as the antilogarithm of the free concentration of the target metal cation in a reference solution of pH = 7.4, where the total concentrations of the metal cation and of the ligand are supposed to be, respectively, 1.0 × 10^−6^ M and 1.0 × 10^−3^ M. Hence, a larger pM value corresponds to a lower concentration of the free metal cation in solution at equilibrium and is associated with a higher capacity of the ligand to form complexes with the target metal cation [35].

**Figure 7:**
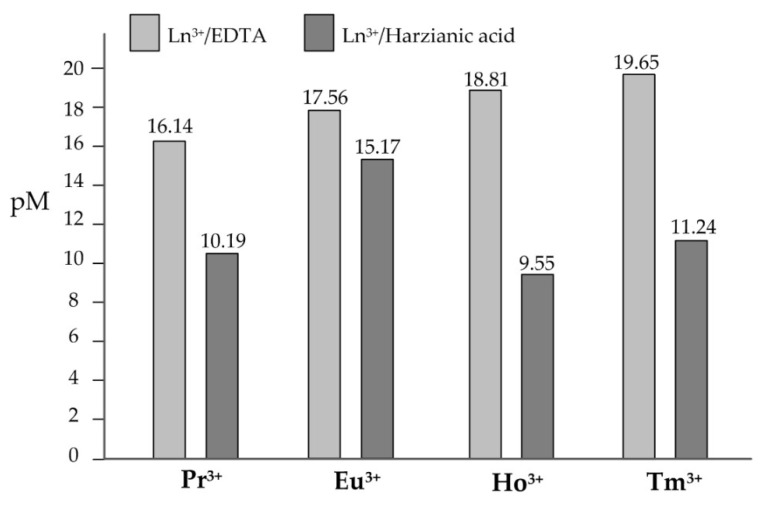
pM values calculated for Pr^3+^/harzianic acid, Eu^3+^/harzianic acid, Ho^3+^/harzianic acid, and Tm^3+^/harzianic acid systems using equilibrium constants determined in this paper in the 0.1 M NaClO_4_/(CH_3_OH + H_2_O 50/50 *w*/*w*) solvent (dark gray bars). Light gray bars represent pM values calculated in water for Ln^3+^/EDTA systems, which are included to serve comparison purposes since EDTA is one of the most popular and efficient chelating agents for metal cations.

The bar plot in Figure 7 shows pM values for the four Ln^3+^/harzianic acid systems considered calculated by using the formation constants determined in this study (dark gray bars). By inspecting Figure 7 we can immediately conclude that harzianic acid is by far a more efficient ligand for Eu^3+^ than for the other three Ln^3+^ cations.

In Figure 7 we also have included (light gray bars) the pM values for Ln^3+^-EDTA systems in water calculated by using data from the literature [36], since EDTA is the most popular and one of the most efficient chelating agents for metal cations and, by consequence, is a very convenient touchstone against which judging the efficiency of other ligands. Evidently, on the basis of the pM criterion, EDTA is by far a more effective ligand than harzianic acid for Tm^3+^, Pr^3+^, and Ho^3+^ but to a very moderate extent more efficient for Eu^3+^.

### 2.3. Ecotoxicity Tests on Raphidocelis subcapitata, Daphnia magna, and Aliivibrio fischeri

Contamination of water bodies is of special toxicological significance because aquatic systems provide humans with food and water through which rare-earth elements can enter the human body [3,14]. Yet, compared to other REEs, such as gadolinium, lanthanum, and cerium, there are very few papers investigating the aquatic toxicity of praseodymium, europium, holmium, and thulium [37]. In general, the toxicity of these elements was assessed using single organism toxicity tests rather than a battery of tests approach that is to be preferred in order to avoid potential biases obtained using single species toxicity assays and in view of the development of REEs water quality guideline values.

Therefore, to fill the gap, in this work we have engaged ourselves in the investigation of the toxicity of Pr^3+^, Eu^3+^, Ho^3+^, and Tm^3+^ toward a suite of three organisms representative of different levels of biological organization and trophic levels including *D. magna*, *R. subcapitata,* and *A. fischeri*. The components of the aquatic ecotoxicity battery applied are *D. magna* acute immobility test [38], *R. subcapitata* chronic algal growth inhibition test [39], and *A. fischeri* acute luminescence inhibition test [40], described in more detail in the experimental section.

In order to determine the median effective concentration (EC50) of each Ln^3+^ cation, the bioassay battery was performed at different concentrations of the Ln^3+^ cations ranging from about 0.1 mM to 1 mM. Results are summarized in Table 4.

Among the selected organisms, *D. magna* seems to be the most sensitive, showing the lowest EC50 values for all Ln^3+^ cations. As can be seen from Table 4, for *D*. *magna* the toxicity of Ln^3+^ cations declines in the order Eu^3+^ ≅ Pr^3+^ > Tm^3+^ > Ho^3+^.

For *R. subcapitata*, EC50 toxicity declines from europium to holmium in a slightly different order (i.e., Eu^3+^ ≅ Tm^3+^ > Pr^3+^ > Ho^3+^).

Interestingly, at the lowest level of biological organization, the order of toxicity of Ln^3+^ cations is almost reversed. In fact, for *A. fischeri* toxicity of Ln^3+^ cations declines from thulium to praseodymium and europium and holmium, which, respectively, seem to be the most toxic and less toxic elements for *D. magna* and *R. subcapitata*, occupy the intermediate positions.

Nevertheless, based on EC50 values integrated over the test battery, displayed in the last column of Table 4, the following toxicity relationship can be established: Tm^3+^ > Eu^3+^ > Ho^3+^ > Pr^3+^.

EC50 values of Eu^3+^, Ho^3+^, and Tm^3+^ toward *D. magna, R. subcapitata,* and *A. fischeri* could not be compared with previous studies because, to the best of our knowledge, this is the first study in which their ecotoxicological effects toward the above bioindicator organisms were investigated. In fact, biotoxicity data of these elements were previously reported only for sea urchins [41,42] and marine algae [43]. Only the EC50 value of Pr^3+^ toward *D. magna* is already available in the literature [44,45], and the reported value (EC50 = 0.064 mM) is in reasonable agreement with the value (EC50 = 0.027 mM) determined in this study, taking into account the many biological and technical variables which can affect the underlying bioassays.

In our previous work [31], we reported the EC50 of gadolinium determined by applying the same suite of bioassays. For *D. magna*, *R. subcapitata,* and *A. fischeri*, EC50 of Gd^3+^ cation is, respectively, 1.1 µM, 3.5 µM, and 2.6 µM. Therefore, so far, gadolinium seems to be by far the most toxic REE, and this fully justifies the great concern raised by the diffusion of this element in the environment. Moreover, we also demonstrated that the toxicity of gadolinium to *D. magna*, *R. subcapitata,* and *A. fischeri* is substantially lowered if these organisms are exposed to comparable concentrations of harzianic acid and gadolinium. Mitigation of gadolinium toxicity by harzianic acid takes place very likely through complex formation (masking) since harzianic acid is a very efficient ligand for gadolinium (pM = 14.13 for the system Gd^3+^-harzianic acid).

Within this framework, it can be predicted that the beneficial effects of harzianic acid should extend to all Ln^3+^ cations considered in this study and especially to Eu^3+^ which is the most efficiently complexed.

Unfortunately, because of technical difficulties, this prediction can hardly be verified experimentally. Difficulties arise from the irreconcilable restrictions imposed on the bioassays to be performed by the relatively large EC50 values exhibited by Ln^3+^ cations considered in this study, by the low solubility in water of harzianic acid, and by the fact that harzianic acid is itself toxic to the tested bioindicator organisms. In fact, EC50 values of harzianic acid toward *D. magna*, *R. subcapitata,* and *A. fischeri* range from 1.2 µM to 3.5 µM, and the no observable effect concentration, EC10, is estimated to be about 0.7 µM [31].

For example, suppose that we plan toxicity tests to demonstrate that the formation of the ${\mathrm{EuL}}_{2}^{-}$ complex of europium can reduce the toxicity of Eu^3+^ cation. Bioassays could be performed by exposing the test organisms first to 0.2 mM Eu^3+^ and then to 0.2 mM Eu^3+^ + 0.2 mM harzianic acid. The relatively high 0.2 mM concentration of Eu^3+^ (or other Ln^3+^ cation) to be employed is required to induce high toxicological effects and is a direct consequence of the high EC50 values reported in Table 4. On the other side, the relatively high 0.2 mM concentration of harzianic acid is needed to convert a substantial fraction of europium to its complex and is a direct consequence of the relatively high Eu^3+^ concentration which unavoidably must be employed to conduct significant ecotoxicological tests. Under such conditions, the risk that the free concentration of harzianic acid in the medium exceeds the no-effect level (0.7 µM) is considerable and cannot be evaluated a priori. In any case, to perform such experiments, the preparation of an aqueous medium fortified with 0.2 mM Eu^3+^ + 0.2 mM harzianic acid is an unavoidable preliminary step. Unfortunately, replicated attempts to prepare a stable medium containing millimolar concentrations of any of the Ln^3+^ cations considered in this study and harzianic acid failed because of the low solubility of harzianic acid in water. Furthermore, the addition of an organic solvent (e.g., methanol) to enhance the solubility of harzianic acid is impracticable because the organic solvent would potentially bias the tests.

The above problems vanish for gadolinium since, as noted above, concentrations of a few µM of Gd^3+^ are sufficient to induce intense toxicological effects toward *D. magna*, *R. subcapitata,* and *A. fischeri*. Under such conditions, very low concentrations of harzianic acid are needed to convert a substantial fraction of Gd^3+^ to its complexes with harzianic acid and to achieve a measurable toxicity reduction effect. For instance, by exposition of *D. magna*, *R. subcapitata,* and *A. fischeri* to 3.7 µM Gd^3+^ + 0.7 µM harzianic acid, we achieved a toxicity reduction ranging from ~20% to ~25%, with respect to exposition to 3.7 µM Gd^3+^; this is a very remarkable effect considering that it was achieved with a harzianic acid to gadolinium ratio of only about 0.7/3.7 ≅ 0.2 which was selected in order to keep harzianic acid concentration at the no-effect level.

However, the very remote possibility may exist that toxicity reduction effects are not due to the masking of the rare-earth cations by complex formation reactions with harzianic acid but to an unexpected biological mechanism requiring very low concentrations of harzianic acid itself, or even of the Ln^3+^/harzianic acid complexes, to be triggered.

**Figure 8:**
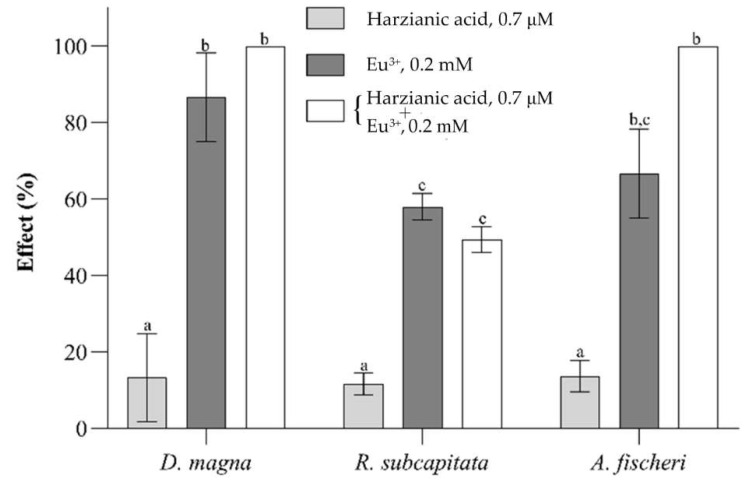
Results of ecotoxicity bioassays performed on *D. magna* (acute immobility test), *R. subcapitata* (chronic algal growth inhibition test), and *A. fischeri* (acute luminescence inhibition test) exposed, respectively, to 0.7 µM harzianic acid, 0.2 mM Eu^3+^, and (0.2 mM Eu^3+^ + 0.7 µM harzianic acid). Data are reported as mean ± SD (*n* = 3). Letters (a–c) indicate significant differences between treatments; the level of significance was set at α = 0.05 (ANOVA).

In order to verify this hypothesis, the battery of bioassays was performed by exposing *A. fischeri*, *R. subcapitata*, and *D. magna* to 0.2 mM Eu^3+^ + 0.7 µM harzianic acid and to 0.2 mM Eu^3+^; results are exposed in Figure 8.

Under the conditions of Figure 8, for simple stoichiometric reasons, only a very low fraction of europium (less than 1%) can be converted to its complex in the 0.2 mM Eu^3+^ + 0.7 µM harzianic acid medium, and, as expected, there is no significant toxicity reduction effect by harzianic acid.

## 3. Materials and Methods

### 3.1. Reagents and Their Analysis

Stock solutions of tripositive rare-earth cations were prepared by dissolving their high-purity oxides in concentrated hydrochloric or perchloric acids (Merck, Darmstadt, Germany). The exact concentration of each metal solution was determined as described by Kolthoff et al. [46]. The solvent, 0.1 M NaClO_4_/(CH_3_OH + H_2_O 50/50 *w*/*w*), was prepared by dissolving NaClO_4_ (Sigma-Aldrich, Saint Louis, MO, USA) in a mixture 50/50 *w*/*w* of CH_3_OH and H_2_O.

The harzianic acid used in this study was extracted from cultures of a marine-derived strain (L1) of *Trichoderma pleuroticola* as reported by De Tommaso et al. [29]. Briefly, the fungal strain was grown in 1 L Erlenmeyer flasks containing 500 mL of potato dextrose broth (PDB, Himedia, Einhausen, Germany) and kept in darkness at 25 °C for 3 weeks. After the incubation period, culture filtrates were obtained by filtering the culture through filter paper (Whatman, Maidston, UK). Then, the culture filtrate of *T. pleuroticola* was acidified with 2 N HCl and exhaustively extracted with ethyl acetate (EtOAc). After solvent evaporation under reduced pressure, the crude extract was dissolved in chloroform and extracted with a saturated aqueous solution of NaHCO_3_. The aqueous phase was acidified and extracted with EtOAc to obtain a residue identified as harzianic acid by comparing the NMR data with previous reports [47].

### 3.2. HPLC–ESI- Q-TOF Analyses

Solutions were prepared by mixing 500 µL of 2 mM aqueous solution of each Ln^3+^ cation (Pr^3+^, Eu^3+^, Ho^3+^, Tm^3+^) chloride or perchlorate salts and 500 µL of harzianic acid 1 mg mL^−1^ in methanol. Then, 7 µL of each sample were injected in an Agilent high-performance liquid chromatography (HPLC) 1260 Infinity Series (Agilent Technologies, Santa Clara, CA, USA) coupled with a quadrupole time-of-flight (Q-TOF) mass spectrometer model G6540B (Agilent Technologies) with a Dual ESI source (Agilent Technologies). The injected volumes were eluted to the mass spectrometer with 0.1% formic acid in acetonitrile at a flow rate of 0.3 mL·min^−1^. The system operated in positive ion mode and a standard solution of purine and hexakis(1H,1H,3H-tetrafluoropentoxy)phosphazene was infused to obtain the real-time lock mass correction. All parameters and acquisitions were set using the Agilent MassHunter Data Acquisition Software, rev. B.05.01 (Agilent Technologies).

### 3.3. Preparation of Test Solutions for CD and UV-Vis Spectrophotometric Measurements

Acquisition workflow for CD and UV-Vis measurements on solutions of rare-earth cations and harzianic acid contemplates the acquisition, for each element, of two groups of CD and UV-Vis spectra on solutions of accurately known pH (=−log[H^+^]) and analytical composition which was specified by the four analytical variables: $C_{\mathrm{Ln}}$ M (molar concentration of rare-earth cation); $C_{H_{2}L}$ M (molar concentration of harzianic acid); $C_{H}$ M (analytical concentration of HClO_4_); and $C_{\mathrm{OH}}^{\;}$ M (analytical concentration of NaOH).

In fact, to help in the interpretation of spectra, solutions in each group were planned to have the same ligand-to-metal ratio (i.e., $\frac{C_{H_{2}L}}{C_{\mathrm{Ln}}}$= *constant*), respectively, very close to 1 for the first group and very close to 2 for the second group (Table 2). To reach this goal, we employed four stock solutions, which, by analysis or preparation, contained accurately known concentrations of Ln(ClO_4_)_3_ or Ln(Cl)_3_ ($C_{\mathrm{Ln}}^{0}$ M), harzianic acid ($C_{H_{2}L}^{\;0}\;M$), HClO_4_ ($C_{H}^{0}\;M$), and NaOH ($C_{\mathrm{OH}}^{0}\;M$) in 0.1 M NaClO_4_/(CH_3_OH + H_2_O 50/50 *w*/*w*); and a potentiometric apparatus constituted by a multi-neck titration vessel equipped with a Metrohm AG (Herisau, Switzerland) 60102–100 pH sensitive glass electrode (GE) and an Ag/AgCl_(s)_/0.1 M NaCl/(0.1 M NaClO_4_/(CH_3_OH + H_2_O 50/50 *w*/*w*) double junction reference electrode (RE).

The experiment started by introducing a fixed volume, $V_{H}$ mL, of the HClO_4_ stock solution in the titration vessel, which was kept in an air thermostat at 25 ± 0.1 °C. This realized a potentiometric cell, GE/Solution/RE, whose potential, $E_{G}\;\mathrm{Volt}$, under the present conditions, can be expressed by the following simple relation (3):

(3) $$E_{G}\left(\mathrm{Volt}\right)=E_{G}^{0}\left(\mathrm{Volt}\right)+Slope\cdot \log\left[H^{+}\right]$$

In order to evaluate the calibration constants $E_{G}^{0}$ and *Slope* in Equation (3), the solution in the potentiometric vessel was alkalimetrically titrated by stepwise addition of accurately measured volumes of the $C_{\mathrm{OH}}^{0}$ M stock solution of NaOH. In all experiments, the alkalimetric titration ended when the same total volume, $V_{\mathrm{OH}}$, of NaOH solution had been added, and the solution in the potentiometric vessel had attained a fixed volume equal to $\left(V_{H}+V_{\mathrm{OH}}\right)$ mL. After the alkalimetric titration, accurately measured volumes of the $C_{\mathrm{Ln}}^{0}$ M solution of Ln^3+^ and of the $C_{H_{2}L}^{0}$ M solution of harzianic acid were added to the $\left(V_{H}+V_{\mathrm{OH}}\right)$ mL of solution in the titration vessel. The added volumes of harzianic acid and metal solutions determined the ligand-to-metal ratio in the resulting solution. After this, the solution was brought to its final pH by adding a measured volume of the $C_{\mathrm{OH}}^{0}$ M solution of NaOH. The volume of NaOH solution added in this step determined the values of $C_{\mathrm{Ln}}$, $C_{H_{2}L}$ and pH in the final solution, which would be used for analysis with the CD and UV-Vis spectrometers; obviously, it did not change the ligand-to-metal ratio.

Subsequently, sufficient time was allowed for chemical equilibrium to be established and for the glass electrode potential, $E_{G}$, to achieve a constant value, which persisted for at least 15 min within ±0.1 mV. Thus, the free proton concentration of the solution in the titration vessel was readily calculated from Equation (4), and the measured $E_{G}$ as follows:

(4) $$E_{G}=E_{G}^{0}+Slope\cdot \log\left[H^{+}\right]\to \;\mathrm{pH}=-\log\left[H^{+}\right]=\frac{E_{G}^{0}-E_{G}}{Slope}$$

Finally, appropriate volumes of solution were withdrawn from the titration vessel and submitted to CD and UV measurements at 25.0 °C.

By this procedure, using fixed volumes of the $C_{\mathrm{Ln}}^{0}$ M stock solution of Ln^3+^ and the $C_{H_{2}L}^{0}$ M stock solution of harzianic acid for each group of CD and UV-Vis measurements, the ratio $C_{H_{2}L}/C_{\mathrm{Ln}}$ was kept the same in each group for each element.

UV-Vis spectra were recorded by Cary model 5000 Spectrophotometer by Varian C. (Palo Alto, CA, USA), from 200 to 600 nm (optical path 0.2 cm) at 25.0 °C, under a constant flow of nitrogen.

The far UV–CD spectra were recorded with a JASCO CD spectrometer model J-715 (Tokyo, Japan), from 250 to 500 nm (optical path 0.2 cm) at 25.0 °C, under a constant flow of nitrogen.

### 3.4. Ecotoxicity Tests

All ecotoxicity tests were carried out according to ISO guidelines towards the following organisms: *A. fischeri*, *D. magna*, *R. subcapitata*.

Sterile aqueous solutions with different concentration of Pr^3+^, Eu^3+^, Ho^3+^, or Tm^3+^ ranging from 0.0001 M to 0.001 M were prepared in ISO medium [38], in order to determine for each Ln^3+^ the median effective concentration (EC50).

To assess the toxicity of europium in presence of the ligand, a sterile solution containing 0.2 mM of Eu^3+^ (concentration inducing high toxicological effects) and 0.7 µM of harzianic acid (concentration corresponding to EC10, [31]) was prepared in ISO medium [38].

The *D. magna* acute immobility test was performed according to ISO 6341:2012 [38]. Five individuals (<24 h old) were incubated in 10 mL of test media in test vessels at 20 °C (±1 °C) in darkness for 24 h. The number of immobilized or dead individuals was checked after 24 h.

The *R. subcapitata* algal inhibition test was carried out according to ISO 8692:2012 [39]. Test organisms were cultured in ISO medium in a light-dark cycle (16 h to 8 h) at 24 °C. The light intensity was 6000 lux. The test was carried out in 24-well plates and the initial cell concentration was 10^4^ cells mL^−1^. After 72 h of exposure, the growth was measured spectrophotometrically at 670 nm and growth rate was calculated by normalizing the cell density to control groups (incubated without the presence of Ln^3+^ cations).

The *A. fischeri* luminescence inhibition test was performed according to ISO11348-3:2007 [40] using commercially available BioTox™ WaterTox™ Standard Kit (EBPI, Ontario, Canada). Controls and dilutions were prepared in 2% NaCl in phosphate buffer. After rehydration of freeze-dried bacteria and exposure of 30 min, the luminescence was measured with a Microtox^®^ analyzer (Model 500, AZUR Environmental) at 15 °C.

Within each ecotoxicological test, two controls and five dilutions of the tested Ln^3+^ cation were used in triplicates.

Data were expressed as effect (%) +/− standard deviation. EC50 were expressed as mean values and the corresponding 95% confidence limit values. Differences between treatments were assessed via two-way analysis of variance (ANOVA) after the verification of normality. The post hoc Tukey’s test accounted for differences within groups setting the statistical significance at α = 0.05. Statistical analysis was carried out using XLSTAT (Addinsoft, Paris, France) and GraphPad Prism (GraphPad, San Diego, USA).

## 4. Conclusions

Complex formation equilibria of the tripositive cations of praseodymium, europium, holmium, and thulium with *Trichoderma* secondary metabolite harzianic acid (H_2_L) in a 0.1 M NaClO_4_/(CH_3_OH + H_2_O 50/50 *w*/*w*) solvent have been investigated and the formation constants of the detected complexes (i.e., PrL^+^, HoL^+^, ThL^+^, and ${\mathrm{EuL}}_{2}^{-}$) determined.

Results of the present investigation can be combined with results from our previous investigation concerning lanthanum, neodymium, samarium, and gadolinium tripositive cations to obtain a picture of interactions of harzianic acid with rare-earth cations extending over 8 of 17 REEs. For instance, based on the pM criterion, the efficiency of harzianic acid as a ligand for tripositive rare-earth cations so far investigated declines in the order: La^3+^(pM = 17.44) > Nd^3+^(pM = 15.46) > Eu^3+^(pM = 15.17) > Gd^3+^(pM = 14.13) > Sm^3+^(pM = 13.65) > Tm^3+^(pM = 11.24) > Pr^3+^(pM = 10.19) > Ho^3+^(pM = 9.55).

Since few field studies, partially with conflicting results, have as yet been performed, a battery of bioassays, employing a suite of bioindicators, was applied to evaluate the aquatic toxicity of the four targeted cations. It is shown that not all organisms reacted in the same way to the Tm^3+^, Eu^3+^, Ho^3+^, and Pr^3+^ applied (see EC50 values in Table 4). Furthermore, based on the evaluated median effective concentration (EC50), Tm^3+^, Eu^3+^, Ho^3+^, and Pr^3+^ appear to be much less toxic to test species than Gd^3+^.

## Figures and Tables

**Table 1:** Most abundant ions in high-resolution mass spectra (HRMS) acquired by HPLC-ESI-HRMS on solutions of harzianic acid and Pr^3+^, Eu^3+^, Ho^3+^, and Tm^3+^ rare-earth cations.

Ion	Observed *m/z* of Main Isotopic Peak	Formula	Exact Mass
**Harzianic acid + EuCl_3_**			
[H_2_L + H]^+^	366.1921	C_19_H_28_NO_6_	366.1917
[H_2_L + Na]^+^	388.1730	C_19_H_27_NO_6_Na	388.1736
[2H_2_L-2H + Eu]^+^	879.2714	C_38_H_52_N_2_O_12_Eu	879.2719
**Harzianic acid + HoCl_3_**			
[H_2_L + H]^+^	366.1915	C_19_H_28_NO_6_	366.1917
[H_2_L + Na]^+^	388.1734	C_19_H_27_NO_6_Na	388.1736
[2H_2_L-2H + Ho]^+^	893.2806	C_38_H_52_N_2_O_12_Ho	893.2823
**Harzianic acid + PrCl_3_**			
[H_2_L + H]^+^	366.1919	C_19_H_28_NO_6_	366.1917
[H_2_L + Na]^+^	388.1732	C_19_H_27_NO_6_Na	388.1736
[2H_2_L-2H + Pr]^+^	869.2606	C_38_H_52_N_2_O_12_Pr	869.2597
**Harzianic acid + Tm(ClO_4_)_3_**			
[H_2_L + H]^+^	366.1922	C_19_H_28_NO_6_	366.1917
[H_2_L + Na]^+^	388.1736	C_19_H_27_NO_6_Na	388.1736
[2H_2_L-2H + Tm]^+^	897.2870	C_38_H_52_N_2_O_12_Tm	897.2862

**Table 2:** Summary of analytical composition and pH of solutions of harzianic acid and, respectively, of Pr^3+^, Eu^3+^, Ho^3+^, and Tm^3+^ in the 0.1 M NaClO_4_/(CH_3_OH + H_2_O 50/50 *w*/*w*) solvent employed to collect CD and UV-Vis spectrophotometric data. $C_{H_{2}L}$ = analytical molar concentration of harzianic acid; $C_{\mathrm{Ln}}\;$ = analytical molar concentration of rare-earth cation; $C_{H_{2}L}/C_{\mathrm{Ln}}$ = analytical ligand to rare-earth cation ratio; pH = −log[H^+^]. All CD and UV-Vis spectra were acquired by employing 0.2 cm optical path quartz cuvettes.

RE Cation	$C_{H_{2}L}/C_{Ln}$	$C_{Ln}\left(pH\right),\;M$
Pr^3+^	0.990	2.42 × 10^−4^ (2.60); 2.23 × 10^−4^ (3.09); 2.17 × 10^−4^ (3.55); 2.07 × 10^−4^ (7.41), 1.96 × 10^−4^ (8.13).
	1.978	0.657 × 10^−4^ (2.63); 0.611 × 10^−4^ (3.12); 0.593 × 10^−4^ (3.67); 0.585 × 10^−4^ (5.29); 0.581 × 10^−4^ (7.01); 0.571 × 10^−4^ (8.08).
Eu^3+^	1.044	1.58 × 10^−4^ (2.25); 1.40 × 10^−4^ (2.74); 1.35 × 10^−4^ (3.28); 1.33 × 10^−4^ (3.80); 1.32 × 10^−4^ (5.12); 1.29 × 10^−4^ (7.79); 1.22 × 10^−4^ (10.47).
	1.980	0.657 × 10^−4^ (2.42); 0.607 × 10^−4^ (3.03); 0.587 × 10^−4^ (6.20); 0.578 × 10^−4^ (7.58); 0.549 × 10^−4^ (8.41).
Ho^3+^	0.995	0.912 × 10^−4^ (2.77); 0.867 × 10^−4^ (3.19); 0.849 × 10^−4^ (3.73); 0.842 × 10^−4^ (4.19); 0.809 × 10^−4^ (8.84).
	1.982	0.644 × 10^−4^ (2.32); 0.607 × 10^−4^ (2.44); 0.569 × 10^−4^ (2.62); 0.519 × 10^−4^ (3.22); 0.504 × 10^−4^ (4.09); 0.497 × 10^−4^ (6.82); 0.492 × 10^−4^ (7.72).
Tm^3+^	1.037	1.66 × 10^−4^ (2.56); 1.53 × 10^−4^ (3.02); 1.47 × 10^−4^ (3.87); 1.46 × 10^−4^ (4.34); 1.44 × 10^−4^ (5.31); 1.43 × 10^−4^ (6.55); 1.38 × 10^−4^ (10.16).
	2.000	0.702 × 10^−4^ (2.64); 0.649 × 10^−4^ (3.09); 0.630 × 10^−4^ (3.56); 0.612 × 10^−4^ (7.36); 0.605 × 10^−4^ (7.94); 0.591 × 10^−4^ (10.29).

**Table 3:** Summary of Ln^3+^/harzianic acid (H_2_L) formation constants. **σ** denotes the estimated standard deviation.

Ln^3+^	Reaction	Log (Formation Constant) ± 3*σ*
Pr^3+^	Pr^3+^ + L^2^^−^ ⇌ PrL^+^	$\log\beta_{110}\;$= 7.20 ± 0.07
Eu^3+^	Eu^3+^ + L^2^^−^ ⇌ EuL^+^ Eu^3+^ + 2 L^2^^−^ ⇌ EuL_2_^−^	$\log\beta_{110}\;$< 3.8 $\log\beta_{120}\;$= 15.19 ± 0.13
Ho^3+^	Ho^3+^ + L^2^^−^ ⇌ HoL^+^	$\log\beta_{110}$ = 6.56 ± 0.08
Tm^3+^	Tm^3+^ + L^2^^−^ ⇌ TmL^+^	$\log\beta_{110}$ = 8.25 ± 0.07

**Table 4:** Median effective concentration (EC50) of praseodymium, europium, holmium, and thulium for *D. magna* (acute immobility test), *R. subcapitata* (chronic algal growth inhibition test), and *A. fischeri* (acute luminescence inhibition test). EC values are expressed in mM and are provided as averages (*n* = 3); values in brackets represent ±95% confidence limits.

Ln^3+^	EC50 (mM)			Integrated EC50 (mM)
	*D. Magna*	*R. Subcapitata*	*A. Fischeri*	
Pr^3+^	0.027 (0.012−0.100)	0.270 (0.170–0.450)	0.490 (0.170–2.200)	0.787
Eu^3+^	0.025 (0.015–0.038)	0.110 (0.088–0.150)	0.290 (0.100–0.610)	0.425
Ho^3+^	0.200 (0.190–0.390)	0.360 (0.260–0.520)	0.110 (0.045–0.290)	0.67
Tm^3+^	0.063 (0.054–0.072)	0.120 (0.086–0.160)	0.080 (0.017–0.570)	0.263

## Data Availability

The data that support the findings of this study are available from the corresponding author upon reasonable request.

## Sample Availability

Samples of the compound harzianic acid are available from the authors.
